# E-Commerce Marketing Optimization of Agricultural Products Based on Deep Learning and Data Mining

**DOI:** 10.1155/2022/6564014

**Published:** 2022-05-18

**Authors:** Hui Yang, Zhuohang Zheng, Chu Sun

**Affiliations:** ^1^College of Economics and Management, Northeast Agricultural University, Modern Agricultural Development Research Center, Harbin 150030, China; ^2^School of Management, Guangdong University of Education, Guangzhou 510303, China

## Abstract

China Internet plus agriculture was first put forward in 2015 by the Chinese government's work report, laying the foundation for the development of Internet plus agriculture and promoting the rapid growth of e-commerce marketing of agricultural products. The combination of agricultural product marketing and e-commerce effectively reduces the intermediate links of agricultural product sales. Many e-commerce professional villages have sprung up in some rural areas across the country, and the number of rural e-commerce stores has continued to grow. At this stage, rural e-commerce has become a new way of agricultural trade, and rural e-commerce has formed a unique rural e-store. At present, the e-commerce market share of agricultural products in rural stores is very large, and its advantages are favored by the government, scientific research institutions, and agricultural products processing enterprises. However, with the gradual development of rural e-commerce, it has also encountered many difficulties. Based on this point, this study applies deep learning and data mining to optimize e-commerce marketing. First, with the growth of the online scale of agricultural product transaction data, the creation of traditional shallow model cannot meet the needs of online data processing. Therefore, this study decides to use the deep learning theory for optimization. It has excellent performance in the technical fields of big data processing and image and voice processing and has strong construction ability, which can effectively represent the characteristics of the model. Combined with the characteristics of e-commerce agricultural products processing and consumer practice, this study designs and develops a new customer value evaluation model based on data mining and e-commerce agricultural products value characteristics in the field of e-commerce. By combining deep learning and data mining technology, this study applies it to the field of e-commerce, so as to promote the transformation of marketing optimization.

## 1. Introduction

The Internet economy has become a major theme of economic growth in the 21st century. The Internet and related technologies are leading the rapid development of economy and trade, surpassing traditional business activities and giving full play to their greatest advantages in daily life [1]. The emergence of e-commerce has opened a new era, which “destroys” the traditional mode of operation and has a great impact on the production, operation, and marketing of enterprises [2]. No enterprise or industry can ignore the changes brought by e-commerce [3]. Only by conforming to the development of e-commerce business and building a new marketing model we can occupy a strong position in the economic wave of the 21st century and obtain the main advantages of market competition [4]. The development of e-commerce has shortened the distance between enterprises and consumers, greatly reduced the marketing cost of enterprises, and promoted the income growth of enterprises [5]. At the same time, compared with traditional commerce, the convenience of e-commerce aims to stimulate consumer consumption, so as to further increase enterprise sales [6]. At the same time, e-commerce goes beyond the space limit and enables enterprises to reach more consumers and attract more consumer groups [7]. Under such extensive influence, agricultural products must also meet the needs of the development of the Internet and integrate more Internet content into the original marketing model [8]. Based on this point, this study analyzes and optimizes the e-commerce of agricultural products by combining deep learning and data mining technology [9]. Due to the fast growth and variability of transaction data and the weak corresponding relationship between sales factors and sales level, the data preprocessing technology based on fuzzy membership is used to optimize it, so as to further optimize the deep learning algorithm [10]. By adding the decentralized restricted self-coding method, this study proposes a low membership deep learning sales prediction model—super crown model (SICM), which effectively solves the factors affecting product sales and balance level [11]. Then, aiming at the problems of unclear target users and low efficiency, this study carries out data mining on the agricultural product customer data provided by enterprises. To identify potential new business customer groups, the personalized needs of customers are taken into account and the consumption probability of customers is predicted [12]. The process of agricultural products should be sorted out to obtain the best recommendation effect, realize reasonable marketing, effectively improve the performance of agricultural products, or carry out online sales. Through such treatment, we hope to help promote the development of e-commerce for agricultural products [13].

## 2. Related Work

This study proposes an online marketing model of agricultural products based on deep learning—crown model (ICM), which uses two-layer automatic coding network to capture samples [14]. The labeled sample set is used to extract the classification features of unlabeled training samples; BP is used to adjust the parameters of the whole network, get the best parameters of the loss function, and realize the dynamic classification and prediction of online sales of agricultural products [15]. A depth weighted K-means clustering algorithm based on an enhanced depth clustering neural network is proposed to realize telecom customer segmentation [16]. This algorithm solves the problem that the traditional K-means algorithm is sensitive to starting the clustering center, further improves the grouping performance, and can be easily extended to large-scale data [17]. Comparative experiments show that the clustering algorithm is superior to other mainstream clustering algorithms in customer segmentation and can provide strong information support for resource planning operators, which fully shows the practical significance of the system [18]. An improved depth neural network (DNN) model SDNN is proposed in the literature. The parallel calculation based on GPU shows that by establishing the model and implementing the algorithm without affecting the prediction effect, the training time of SDNN prediction model is reduced by about 73.28%, and the efficiency of DNN calculation is further improved [19]. Literature has confirmed that a relu activation function is more suitable for deeper network models than sigmoid activation function, while DNN and SDNN based on the relu activation function are more suitable for modeling complex problems. The literature makes an in-depth study on the development of e-commerce operation mode at home and abroad and expounds on the role and importance of big data in the future development of e-commerce [20]. The future development framework of e-commerce is established based on big data, so as to predict the mode and marketing trend of agricultural products in the future.

## 3. Theoretical Basis

### 3.1. Deep Learning

The research on online sales prediction of agricultural products involves the application of information science pattern recognition in the field of e-commerce. As for the research on commodity sales prediction methods, the research at home and abroad is mainly based on the analysis and comprehensive prediction of commodity sales prediction related methods, such as gray theory, artificial neural network, and time series. Such methods have low prediction accuracy when dealing with a large amount of data, and such models usually lack long-term effectiveness and scalability.

An automatic encoder (AE) was proposed by Rumelhart in 1986. It is a deep learning method with a fast learning ability. Its main purpose is to learn the compressed distribution feature expression of a given data set. Its essence is to use the characteristics of the hierarchical structure of artificial neural network (ANN), use the hierarchical unsupervised self-monitoring pretraining and parameters for system optimization, and extract the hierarchical features from large-scale complex input data, so as to obtain a distributed original data distribution. Feature representation deep learning neural network structure shows the high efficiency of processing high-dimensional big data. A simple AE consists of three components: encoder, decoder, and hidden layer. The biggest difference between it and the traditional neural network, which directly uses the predictive learning results, is that the automatic coding network only pays attention to the hidden parameters of layer weight and does not carry out classification operation.

Assuming that an unlabeled training data sample *V* is input, the encoder maps *V* to the hidden layer *H*. V is converted to H activated by formula (1), and *V* and H satisfy the following formula:

(1) $$\begin{array}{c} h=f_{\theta }\left(v\right)\\ =s_{f}\left(W^{T}v+b_{n}\right). \end{array}$$

Among them, s_f_ is a nonlinear activation function, which usually uses the logic function sigmoid function. It is a common S-shaped function in biology, also known as an S-shaped growth curve. Its definition formula is as follows:

(2) $$\begin{array}{c} S\left(z\right)=\frac{1}{1+e^{-z}}. \end{array}$$

*θ*={*W*, *b*} is the parameter set. *H* reconstructs the input value *V* into *y* through reverse coding, and *H* and *y* satisfy the following formula:

(3) $$\begin{array}{c} y=g\left(h\right)\\ =s_{g}\left(W^{1}h+b\right), \end{array}$$

where *θ*_1_ = {*W*^1^, *b*^1^} is the parameter set and *s*_*g*_ is the decoder activation function. The typical formula (2) is selected. The relationship between parameters *W*^1^ and *W*^*T*^ in equations (1) and (3) is *W*^1^ = *W*^*T*^ The reconstruction error *J*_*AE*_ is reduced by continuously optimizing and updating *θ* and *θ*_1_. *J*_*AE*_ is defined as follows:

(4) $$\begin{array}{c} J_{AE}=\sum_{v\in V}L\left(v,y\right), \end{array}$$

where *V* is the input data set and *L* is the reconstruction error function, and the expression of *L* is as follows:

(5) $$\begin{array}{c} L\left(v,y\right)={\left\|v-y\right\|}^{2}. \end{array}$$

Automatic encoder learning uses a simple identity function to realize the complete reconstruction of the original data, while for complex data structure, it needs to learn in complex nonlinear function, because the self-encoder cannot meet the needs of complex data structure and complex learning nonlinear function. Therefore, most advanced learning rarely uses coding, which effectively improves the stability of the model. Sparse self-coding is to add sparse constraints according to the structural characteristics of artificial neural network on the basis of automatic encoder, so as to use the least number of hidden layer units to express the input characteristics, that is, hidden layer nodes. It is suppressed most of the time and active only a few times, so when the number of hidden layer units is large, the structural part of the input vector is still visible. The main purpose of unsupervised vector learning is to use the sparse vector set of ∅_*i*_ encoder for automatic learning:

(6) $$\begin{array}{c} X=\sum_{i=1}^{k}\alpha_{i}\emptyset_{i}. \end{array}$$

Among them, *i* = 1, 2,…, *k* (*k* is the number of input nodes) and *α*_*i*_ is a linear correlation coefficient.

The average activation value of hidden layer unit *j* is as follows:

(7) $$\begin{array}{c} {\hat{\rho }}_{J}=\frac{1}{m}\sum_{i=1}^{m}\left[a_{j}^{\left(2\right)}\left(x^{i}\right)\right]. \end{array}$$

Among them, *i* = 1, 2,…, *m* (*m* is the number of input nodes, *m* = *k*) and *a*_*j*_^(2)^(*x*^*i*^) represents the activation amount of hidden layer unit when the input is *x*.

The penalty factor is expressed in the form of relative entropy KL:

(8) $$\begin{array}{c} \text{KL}\left(\rho \left\|\;\right.{\hat{\rho }}_{J}\right)=\rho \;\log\frac{\rho }{{\hat{\rho }}_{J}}+\left(1-\rho \right)\log\frac{1-\rho }{1-{\hat{\rho }}_{J}}. \end{array}$$

The general cost function of sparse automatic coding network is as follows:

(9) $$\begin{array}{c} J_{\text{spare}}\left(w,b\right)=J\left(w,b\right)+\mu \sum_{j=1}^{s_{2}}KL\left(\rho \left\|\;\right.{\hat{\rho }}_{J}\right). \end{array}$$

Among them, *J*(*w*, *b*) is the global loss function, *λ* is used to control the importance of the second part of the formula and prevent overfitting, and *μ* is the penalty coefficient. The higher the penalty is, the sparser the result is:

(10) $$\begin{array}{c} J\left(w,b\right)=\left[\frac{1}{m}\sum_{i=1}^{m}J\left(W,b;x^{i},y^{i}\right)\right]+\frac{\lambda }{2}\sum_{l=1}^{n_{l}-1}\sum_{i=1}^{S_{l}}\sum_{j=1}^{S_{l}+1}{\left(W_{ji}^{l}\right)}^{2}. \end{array}$$

Among them, *J*(*W*, *b*; *x*^*i*^, *y*^*i*^) is the unit loss function:

(11) $$\begin{array}{c} J\left(W,b;x^{i},y^{i}\right)=\frac{1}{2}{\left\|h-y\right\|}^{2}. \end{array}$$

The output error is obtained according to formula (11):

(12) $$\begin{array}{c} \delta_{i}^{\left(3\right)}=\frac{\partial }{\partial r_{i}^{\left(3\right)}}\frac{1}{2}{\left\|h-y\right\|}^{2}=-\left(y_{i}-\alpha_{i}^{\left(3\right)}\right)f^{\prime }\left(r_{i}^{\left(3\right)}\right). \end{array}$$

*δ*_*i*_^(3)^ represents the error of the output layer and *α*_*i*_^(3)^ is the activation function:

(13) $$\begin{array}{c} r_{i}^{\left(3\right)}=W_{i}^{\left(2\right)}\alpha_{i}^{\left(2\right)}+b^{\left(2\right)}. \end{array}$$

Thus, the error of hidden layer element is as follows:

(14) $$\begin{array}{c} \delta_{i}^{\left(2\right)}=\left(\left(\sum_{j=1}^{s_{2}}W_{ji}^{\left(2\right)}\delta_{j}^{\left(3\right)}\right)+\mu \left(\frac{-\rho }{{\hat{\rho }}_{J}}+\frac{1-\rho }{1-{\hat{\rho }}_{J}}\right)\right)f^{\prime }\left(r_{i}^{\left(2\right)}\right). \end{array}$$

### 3.2. Multilabel Classification Method of Data Mining

As one of the most commonly used data mining scenarios, the recommendation system can recommend products of interest according to customers' personal needs and consumption habits, improve product order rate, and realize reasonable marketing. Data mining often uses classification algorithms to build recommendation systems. The classification task obtains the classification model based on the known samples in order to classify the unknown samples. Common classification problems usually include two classification and multiclassification. Both of them are single label classification problems; that is, each sample in the training set has a label. If the label set *L* = 2, it is a two classification problem; if *L* > 2, it is a multiclassification problem.

For a given sample *X* and a corresponding set of tags *Y*, *N* (*X*) is used to represent the sample *K* closest to the sample *x.* The main steps of implementing the ml-knn algorithm are as follows:

(1) Calculate the label set as shown in the following equation:
  (15) $$\begin{array}{c} Y=\left\{y_{1},y_{2},\ldots ,y_{j}\right\}. \end{array}$$
A priori probability of each label is calculated as follows:
  (16) $$\begin{array}{c} P\left(H^{L}\right)=\frac{s+\sum_{i=1}^{m}\left[\left[y_{j}\in Y_{i}\right]\right]}{s\times 2+m}, \end{array}$$
where *s* is the smoothing term, usually 1.
(2) Calculate the number of nearest neighbors *N*(*t*) marked *L* to *K* corresponding to each sample in sample set *X*:
  (17) $$\begin{array}{c} C\left(L\right)=\sum_{a\in N\left(t\right)}y_{a}\left(L\right). \end{array}$$
(3) Calculate the conditional posterior probability *P*(*E*_*C*(*L*)_^*L*^*|H*_*b*_^*L*^) of each sample in *N* (*t*).
(4) Calculate the k-nearest neighbor *N* (*x*) of the unknown sample.
(5) Calculate the maximum a posteriori probability of unknown samples:
  (18) $$\begin{array}{c} y=\arg\underset{b\in \left\{0,1\right\}}{\max}p\left(H_{b}^{L}|E_{C\left(L\right)}^{L}\right). \end{array}$$

The label marked with 1 is the prediction result of the classification.

Like KNN, the main problem of ml-knn algorithm is the selection of *K* value. This algorithm is not suitable for big data classification.

Xgboost model is a combination of several decision trees. Adding a tree each time you split a feature is equivalent to adding a new function. Each time a tree is added, it is expected to improve the classification effect, that is, to ensure that the value of the loss function is reduced after adding a new function. However, in the process of adding trees, the number of leaf nodes may be too large and the model may be overfitted. Therefore, a regularization penalty should be added to the loss function to limit the number of leaf nodes. Finally, the objective function of the model consists of loss function and regularization:

(19) $$\begin{array}{c} \text{obj}=\sum_{i}^{n}L\left(y_{i},{\hat{y}}_{i}\right)+\sum_{k}^{n}\Omega \left(f_{k}\right). \end{array}$$

Of which

(20) $$\begin{array}{c} {\hat{y}}_{i}=\sum_{k=1}^{K}f_{k}\left(x_{i}\right),\;f\in F. \end{array}$$

*F* is a set of *K* decision trees, and each *f* is one of them. It is defined as (*x*) = *wp*(*x*). The parameters are leaf node *w* and tree structure *ρ*, where *ρ*(*x*) is the index of leaf node where *x* is located.

### 3.3. Design of Data Mining Steps

Data mining is not a simple process of mining results by establishing a data-driven model but a process of repeated iteration and continuous improvement. Generally speaking, the data mining process is as follows:

 
*Understanding the Project*. First, understand the project requirements from the business perspective, then analyze the project from the technical perspective of data mining, and put forward a preliminary scheme to meet the business requirements by combining theory with practice.
 
*Understanding Data*. After setting the project objectives, it is necessary to sort out the relevant data, then describe and analyze the data, and make a qualitative evaluation of the data quality.
 
*Data Preprocessing*. The original data are processed in the appropriate final data set for modeling processing.
 
*Modeling*. Select appropriate modeling methods and adjust model parameters according to the quality and scope of the data itself. Once the algorithm is selected, the training data set will create a model. In the training process, the relevant parameters of the algorithm are continuously adjusted to obtain the best model. After modeling, use the test data set to test or predict the model.
 
*Evaluation*. Conduct a comprehensive and hierarchical evaluation of the model. The evaluation method should consider the differences between algorithms and application fields and select appropriate evaluation parameters. If the verification results meet the requirements, that is, the model really meets the business requirements, then go to the next mining step. Change the direction of data analysis and make plans.
 
*Model Implementation*. Modeling is not the ultimate goal of mining. After getting an ideal model that meets the business requirements, we also need to understand the business of the model and provide interpretation and guidance to support the actual business application.

## 4. Current Situation of E-Commerce Marketing of Agricultural Products

### 4.1. Investigation Methods

#### 4.1.1. Determination of Sample Size

*Determine the Sample Size*. In the investigation of an e-commerce rural store, the operators of e-commerce rural stores were the main respondents. As there are many types of e-commerce products mainly engaged in agricultural products in rural e-commerce shops in e-commerce villages, in order to ensure the correctness and objectivity of the survey, the author uses the method of multistep sampling to determine the residents of e-commerce village who finally conducted the survey. In this process, the deff design effect is used to realize simple random sampling. The confidence level is set to 0.95, and the sample size is set to 0.5 and 0.3, which are expressed by P and Q, respectively. The error sampling limit is controlled at about 5%, the deff value is 1.3, and the final sample size is 293. In addition, 10 e-commerce villages in a county were selected as the survey items. Due to the influence of various factors, the recovery rate of this survey is not 100%, but 90%, so the actual number of questionnaires is 297/90% = 330. Therefore, in this survey of 10 e-commerce villages in a county, the total scale of 330 e-commerce rural stores is taken as the sample.

#### 4.1.2. Sample Allocation

As the survey is a nonrepeated sampling survey, the sampling units in the first stage are all e-commerce villages in a county (6 towns, 3 ordinary townships, and 1 ethnic township), with a total of 4 streets, 183 administrative villages, 4 neighborhood committees, and 15 communities. Ten e-commerce villages are selected as the survey elements, that is, the sample units in the second stage. In the follow-up distribution of this survey, an appropriate proportion of households, namely, rural online stores, will be selected according to the sample units in the second stage. In the sample, considering the subtle differences in the distribution of rural e-commerce stores, the probability sampling method is selected, and the actual random sampling is carried out according to the proportion of household administrative summary in the total number of rural households. Finally, the total number of households actually received the query, that is, the number of e-commerce rural stores, is taken as the test unit of the third stage.

In addition, this study also conducted separate interviews with e-commerce village partners, Ali operation center, and county government staff, added countermeasures to the questionnaire, and conducted investigation and analysis, making the results more reasonable and scientific.

#### 4.1.3. Sample Selection Method

On the one hand, the second stage sampling of rural local electricity store operators is divided from regions to rural electricity stores. The survey is divided into two parts: t county and administrative village. Each e-commerce rural store is taken as the unit for the general survey. On the other hand, a multistage sampling survey is conducted on local e-commerce rural partners, from regional divisions to administrative villages, then to e-commerce rural stores, and finally, to e-commerce rural partners. In the process of regional division, according to the division of administrative regions, this sampling method makes the distribution of samples more uniform. In the sampling process of administrative villages, a typical sampling survey of 10 administrative villages in t county is adopted to determine a reasonable sampling interval.

#### 4.1.4. Questionnaire Design Method and Process

A total of 20 questions were raised in this questionnaire, including five questions: the operation and management system of rural electric stores, the quality and brand of rural e-commerce products, the logistics and transportation of rural agricultural products, the technical talents of rural electric stores, and the effectiveness of e-marketing of rural electric stores. Among them, there is no special limited investigation on the types of agricultural products in e-commerce rural stores. A total of 330 questionnaires were distributed and 330 were recovered. Under the supervision and guidance of the investigators, there were 330 valid questionnaires, and the recovery rate and effectiveness rate of the questionnaire were 100%.

### 4.2. Data Processing

According to the purpose and content of the study, SPSS version 22.0 analyzes the relevant data and summarizes the research results. The data of the same group were compared with the paired *t*-test samples, and the data of different groups were compared with the sample *t*-test. The significance level was set as *P* = 0.05.

### 4.3. Research Results

**Figure 1:**
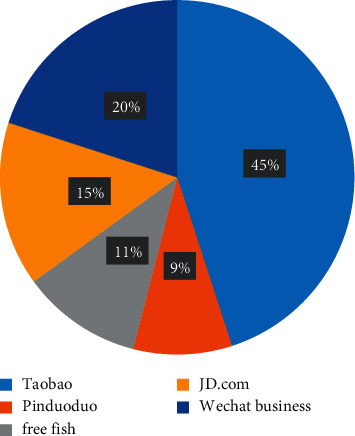
Proportion of sales platforms.

As shown in Figure 1, more than 45% of online store farmers in a county choose Taobao, 9% choose pinduoduo, 11% choose Xianyu, 15% choose Jingdong, and other farmers choose wechat.

More than half of the farmers have opened online stores for 1–3 years and have just entered the online sales team recently. Only a relatively small proportion of online shoppers have been online for more than three years. Most of the employees of the online store are relatives, so the customer service, packaging, and warehousing work are completed internally. Because most of them are self-employed, they rarely recruit foreign personnel. Influenced by the education level and education level, the reputation level of online stores is different. Only 16% of the respondents were crown-level online stores, and only 50% were diamond-level online stores.

**Figure 2:**
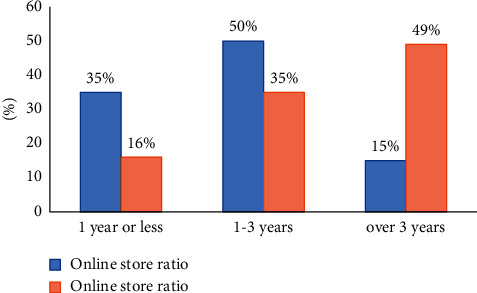
Basic information of online store.

As shown in Figure 2, in the survey of online sales of agricultural products in rural e-commerce, 55.7% of rural e-commerce adopts the wholesale and retail trade of agricultural products, 23.5% of rural e-commerce only adopts the wholesale trade of agricultural products, and 16.7% of rural e-commerce only adopts the retail trade, as listed in Table 1. The results show that in order to protect their own economic interests, rural e-commerce mostly adopts the way of wholesale and retail, while the operation and management of agricultural products in e-commerce have not been fully developed.

In the rural e-commerce agricultural product marketing management survey, up to 59.7% of rural e-commerce store operators said that they could only use a small amount of information technology to achieve governance, and 23.5% of rural e-commerce owners said they could make full use of big data and other advanced information technologies for management. About 16.8% of rural e-commerce operators said that they could not use advanced information technology to achieve information governance, as listed in Table 2, which fully shows that there is a certain lag in the management system of the current e-commerce business model.

**Figure 3:**
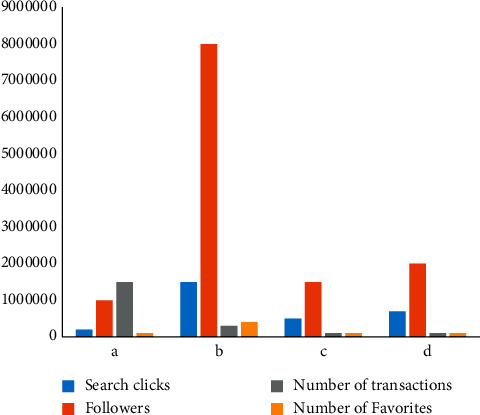
Market scale.

Market attention is one of the main factors affecting business growth. The number of search clicks is proportional to the number of followers, and the higher the number of searches, the more followers. Taking company a as an example, comparing company a with the other three companies in the same industry, we can see the current concern of the nut market in a certain city. Market attention is analyzed from four aspects: the number of search clicks, the number of followers, the number of transactions, and the number of favorites, as shown in Figure 3:

Company a has no obvious advantages in the number of search clicks, followers, and collectors, but it is higher than the other three companies in the number of transactions. Most of the reasons are related to customer service behavior and website interface. Good customer service attitude, short web response time, and website interface have attracted consumers' attention to ensure that customers will not have “running orders.” If consumers are familiar with a brand, they will continue to pay attention to the brand. If there is a demand, the brand will be the first choice, and consumers will conditionally look for the brand and buy it.

**Figure 4:**
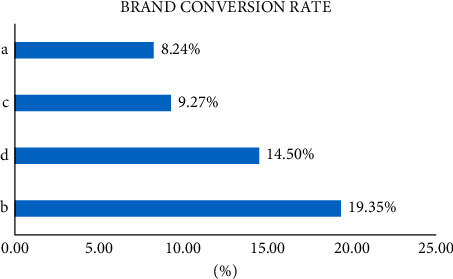
Brand conversion rate.

As shown in Figure 4, the number of search clicks is directly proportional to the number of followers, and the total click-through rate of each promotion and the conversion rate of concerned brands will also increase. Therefore, companies with a large number of collections may not conduct multiple transactions for customers. The survey shows that some consumers will continue to browse stores after collecting goods. If they find products with lower prices, more discounts, and better evaluation, they will give up collecting and focus on another product.

Second, after investigation, the problems of agricultural products existing in rural e-commerce mainly include the high price of agricultural products, poor quality of agricultural products, low brand awareness of agricultural products, few types of agricultural products, and insufficient after-sales service level. Among them, the survey proportion of low quality of agricultural products and low brand awareness of agricultural products ranks first and second, as listed in Table 3. It can be seen that in the process of e-commerce of agricultural products, the quality and brand of e-agricultural products are now the most prominent problems.

Through the survey, it is found that the 330 rural e-commerce stores surveyed rely on the e-commerce marketing income of agricultural products every year, as listed in Table 4.

### 4.4. Problems in E-Commerce Marketing of Agricultural Products

The survey found that rural online stores often have some problems that are unfavorable to the e-commerce marketing of agricultural products, such as poor quality of agricultural products and poor brand construction effect. For example, a survey found that among the operators of Taobao rural stores, 48.6% of the agricultural products of Taobao rural stores were purchased from surrounding farmers, 31% of the agricultural products were directly supplied by suppliers, and only 20.4% of the agricultural products of rural online stores were produced by themselves. Purchasing from farmers often lacks scientific identification and quality control, which is easy to lead to quality problems of agricultural products, while moderate brand promotion activities are only carried out occasionally. Overall, the problems of agricultural products in rural online stores mainly include the high price of agricultural products, poor quality of agricultural products, low brand awareness of agricultural products, few varieties of agricultural products, and low after-sales service level of agricultural products. Therefore, the e-commerce transaction quality and brand construction of agricultural products in rural online stores need to be improved.

## 5. Related Systems and E-Commerce Marketing Optimization Methods of Agricultural Products

### 5.1. System Design

**Figure 5:**
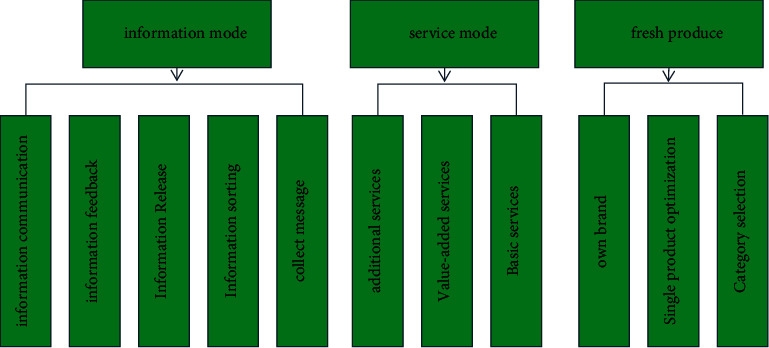
Basic layer operation strategy.

During the operation of the basic layer, it includes information mode, service mode, and products, as shown in Figure 5. Products are taken as the core and information mode and service mode are taken as auxiliary support means. The foundation layer is the most important and basic part of the whole platform, which will play a decisive role in the future development of e-commerce of fresh agricultural products to a certain extent.

The whole field of modern e-commerce is a big data era. The information process of various industries involved in e-commerce usually adopts the “cloud information” mode, including the efficient collection of “cloud information,” the organization of information within the platform, the release of efficient information, information feedback market, and information exchange based on this information.

**Figure 6:**
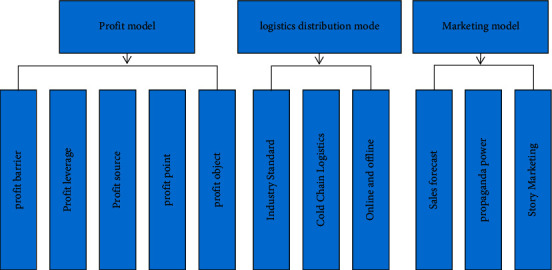
Core layer operation strategy.

As the core part of the e-commerce operation mode of fresh agricultural products, the core layer mainly includes logistics distribution mode, marketing mode, and profit mode, with sales mode as the core, logistics distribution mode as the guarantee, and profit mode as the goal, as shown in Figure 6.

### 5.2. Algorithm Design of Marketing Model

The specific implementation steps of SICM algorithm are as follows:

1. Preprocess the collected online transaction data, which is divided into training data set and test data set.
2. Write the test data set, that is, mark the specific level of sales indicators, which is divided into two parts: one part is used to drive the SICM model and the other part is used to test the performance of the SICM model.
3. Because the online transaction data has the characteristics of large capacity, variability, and high speed, the model determines the number of hidden layers and neurons in each layer in the small-scale automatic coding network through repeated experiments, so as to achieve higher sales and accurate classification.
4. The training data set is used as the input vector of sparse self-encoder network for unsupervised pretraining.
5. In the softmax classifier, labeled and unlabeled data are used as input vectors to drive the classifier.
6. BP is used to improve the parameters of deep learning network to make the parameters reach the global highest level.

When *λ* ≠ 0, the weight value attenuation function is activated, and the weight update rule is as follows:

(21) $$\begin{array}{c} \Delta W_{ij}=-\frac{\eta \;\partial J}{\partial \Delta \;W_{ij}}, \end{array}$$

where *η* is the learning rate. Generally speaking, taking *η* as a small value will lead to gradual error convergence, and *η* is determined by the specific scale of the network. In this study, *η* = 0.05 is selected.

The weight update formula is as follows:

(22) $$\begin{array}{c} \Delta W^{\left(l\right)}=\Delta W^{\left(l\right)}+\nabla_{W\left(l\right)}J\left(W,b;x,y\right),\\ \Delta b^{\left(l\right)}=\Delta b^{\left(l\right)}+\nabla_{b^{\left(l\right)}}J\left(W,b;x,y\right), \end{array}$$

where ∆(*l*) and ∆ *b* (*l*) are the initial values, and the value is 0. The weight is obtained according to the following formulas:

(23) $$\begin{array}{c} W^{\left(l\right)}=W^{\left(l\right)}-\alpha \left[\left(\frac{1}{m}\Delta W^{\left(l\right)}\right)+\lambda W^{\left(l\right)}\right], \end{array}$$

(24) $$\begin{array}{c} b^{\left(l\right)}=b^{\left(l\right)}-\alpha \left(\frac{1}{m}\Delta b^{\left(l\right)}\right). \end{array}$$

### 5.3. Analysis of Experimental Results

Logistic regression is one of the most common models for CTR prediction problems, which is widely used by major companies. Decision tree is the basic model of GBDT. In this experiment, the decision tree model is added. In terms of CTR prediction, the GBDT model is the top noun in major competition platforms. As a representative of integrated learning, GBDT also has a research value. The structure of the neural network model is 2022-1000-2, and the initial weight of the model is random [0, 1].  Table 5 lists the parameter settings for each shallow learning model.

**Figure 7:**
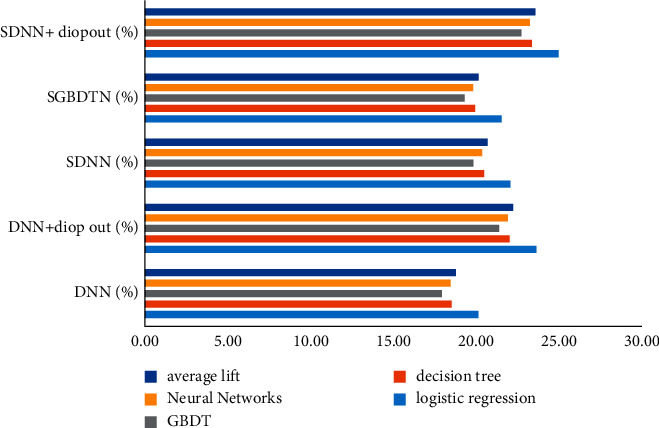
Percentage of relative improvement effect.

The comprehensive comparison results of various methods are listed in Table 6:

Compared with the benchmark classifier of shallow learning, the improvement effect percentage of deep learning is shown in Figure 7.

### 5.4. E-Commerce Marketing Optimization Method of Agricultural Products Based on Relevant Systems

Big data are used to improve the user experience. Big data can play a more humanized role in precision marketing, improve the user experience from obtaining product information to completing the purchase, and make users feel safer and more confident in the whole consumption process. Using big data to let consumers identify brands, the thinking stage is the cornerstone of cultivating brand loyalty. In real life, when buying products or services, most people tend to choose brands they are familiar with, rather than brands they have never heard of. Therefore, brand building is very important. Integrating advertising, business news, etc., and using big data can effectively improve brand awareness, such as sending messages to web pages, consumer shopping carts, and other places. At this stage, the relationship between enterprises and customers is relatively weak. If the products, services, or marketing activities of competitive products are more attractive, such consumers are more likely to use competitive products. Using big data to analyze the consumer identification stage, on the premise of identifying brand customers, customers decide whether to buy or not. At the first purchase, it indicates that the customer is familiar with the products of the brand. However, in the process of purchase, customers constantly waver and question their choice. If they can buy products at the most satisfactory price, after the first purchase, customers will see the value of products or services and master the data to be evaluated in their mind. Using big data to evaluate potential customers can take reasonable measures to win the favor of consumers when they are dissatisfied and let them buy and consume again and again. If the customer is satisfied with the product or service they bought for the first time, they will have a preference for the product or service and buy it again.

## 6. Conclusion

The rapid development of e-commerce has affected various social industries, from traditional electronic products and clothing to daily necessities and food. At present, more and more industries are affected by the Internet, and the depth of the impact is becoming greater and greater. At the same time, the state is also gradually increasing its support for e-commerce of agricultural products. Fresh green products and delicious food from all over the country can be delivered to the door through the e-commerce platform. The convenience of e-commerce of agricultural products can stimulate consumers to consume and further increase the sales of rural online stores. This study takes the data of rural online stores in a county as the research object, adopts the methods of literature retrieval, case analysis, problem investigation, and data analysis, and puts forward an optimization scheme of e-commerce agricultural products marketing based on relevant theories, in combination with in-depth learning and data mining technology, thus it will be helpful to e-commerce agricultural product marketers.

## Figures and Tables

**Table 1:** Forms of online sales of agricultural products by rural e-commerce.

Form	Proportion (%)
Wholesale and retail of agricultural products	55.7
Wholesale form of agricultural products	23.5
Agricultural products retail method	16.7

**Table 2:** Technical management of e-commerce marketing of rural agricultural products.

Technical management	Proportion (%)
It can make full use of advanced information technology such as big data to realize information management	23.5
Some information technology can be used to realize information management	59.7
Completely unable to use advanced information technology to achieve information management	16.8

**Table 3:** Problems of rural e-commerce agricultural products.

Existing problems	Proportion (%)
High agricultural prices	26.3
Low-quality agricultural products	65.8
Low brand awareness of agricultural products	78.4
Lack of varieties of agricultural products	36.4
Insufficient after-sales service of agricultural products	53.7

**Table 4:** Annual income of rural e-commerce relying on agricultural product marketing.

Income	Proportion (%)
Below 100,000 yuan	35
110,000–200,000 yuan	36
210,000–300,000 yuan	21
310,000 yuan	8

**Table 5:** Parameters of shallow learning model.

Shallow learning model	Parameter item	Parameter settings
Logistic regression	Maximum number of iterations	200
	Stop iteration error	l*e2* − 4
	Regular term	L2

Decision tree	Split standard	Gini
	Minimum number of split samples	2

GBDT	Split standard	Gini
	Number of decision trees	200
	Learning rate	0.1

Neural networks	Number of hidden layer nodes	1000
	Activation function	Logistic
	Maximum number of iterations	200

**Table 6:** Comparison results of various methods.

---	AUC
Logistic	0.5961
DT	0.6033
GBDT	0.6072
NN	0.6028
DNN	0.7161
DNN + dropout	0.7361
SDSS	0.7277
SGBDTN	0.7230
SDNN + dropout	0.7466

## Data Availability

The data used to support the findings of this study are available from the corresponding author upon request.
